# Value of videoroscopy in the detection of alterations of Actinic Cheilitis and the selection of biopsy areas

**DOI:** 10.4317/medoral.20297

**Published:** 2015-02-07

**Authors:** Ana-Maria Miranda, Thiago Ferrari, Taiana Leite, Tabata Domingos, Karin Cunha, Eliane Dias

**Affiliations:** 1 DDS, MS, PhD. DDS. DDS, MS student. MD, MS student. DDS, MS, PhD. MD, MS, PhD. Department of Pathology, School of Medicine, Fluminense Federal University - UFF - Niterói - Rio de Janeiro - Brazil

## Abstract

**Background:**

To demonstrate the value of videoroscopy in identifying lesions and alterations not seen by oroscopy and to select the area for biopsy.

**Material and Methods:**

Eighty patients were subjected to anamnesis, physical exam, videoroscopy exam, toluidine blue test and biopsy. A diagram of the lips was created to record the exact location where the lesion was found.

**Results:**

Physical exam identified 287 lesions, and videoroscopy identified 587 lesions; erythema and white lesions were the most common lesions associated with actinic cheilitis. Of the 59 performed biopsies, 32 (52.4%) cases were identified by videoroscopy that showed lesions that were not detected during physical examination.

**Conclusions:**

The establishment of a diagram of the lip permitted registration of the precise location of the lesion. Videoroscopy was effective in locating lesions not seen by oroscopy. Both videoroscopy and the diagram of the lips allowed for better and earlier diagnosis and better patient follow-up for those with actinic cheilitis.

**Key words:** Actinic cheilitis, potentially malignant disorder, videoroscopy, dermatoscopy, lip. oroscopy, diagram of lip.

## Introduction

Among all of the pathological processes that occur in the lips, the most frequent and most important is Actinic Cheilitis (AC), which is caused by chronic exposure to solar radiation. It is considered a potentially malignant disorder because it can progress to Squamous Cell Carcinoma (SCC) of the lip. (1,2) AC is usually asymptomatic, and in most clinical cases, the lips exhibit diffuse lesions with various simultaneous changes that render it difficult to choose the best place to perform a biopsy. (3) The clinical aspects are mainly represented by flaking, dryness, white lesions, ulcers, blurring between the border of the vermilion and the skin and the presence of other lesions. (1,3-5) Diagnosis, proper treatment and patient follow up are essential for preventing the emergence of SCC of the lip or for its early diagnosis (4).

Videoroscopy is a clinical method of examining the oral mucosa through the magnification of an image under adequate lighting with the purpose of evaluating modifications of the mucosal surface. This technique helps the clinician choose an area for biopsy; additionally, the storage of the captured image allows subsequent comparisons for efficient follow up of patients (6). Thus, the aim of this study was to demonstrate the value of videoroscopy in identifying lesions and changes present in AC patients that could not be observed via oroscopy and to demonstrate its usefulness as a tool in the selection of the biopsy area in patients with AC.

## Material and Methods

Samples were obtained from 80 patients who were clinically diagnosed with AC and exhibited no other associated lesion. These patients sought treatment from the Clinic of Oral Diagnosis at Antonio Pedro University Hospital and were treated between August 2010 and December 2012. All patients accepted as participants in the study signed an informed consent waiver. Patients were then interviewed to collect clinical history, and physical examinations of the lips were subsequently performed. The macroscopic lesions were first evaluated through oroscopy, without any type of augmentation resource, followed by documentation with a digital camera (Canon Rebel XTI and Canon Objective EFS 18 - 55 mm, Canon, Japan). After this, videoroscopy, a visual exam that uses an image amplification resource, commonly achieved by oral videocameras, was performed, in our case, using a digital microscope (AVANTSCOPE MAXX™®, Brazil) at 50x magnification. The microscope was connected to a laptop loaded with specific software to enable real-time observation and storage of the captured images.

**Figure 1:**
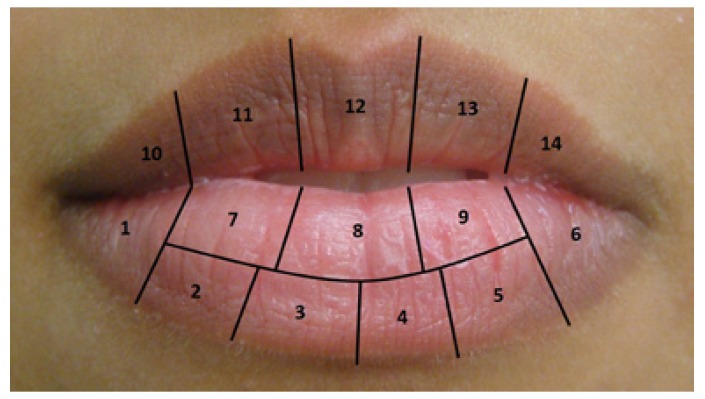
Diagram of the lip: Proposed map of the lips as a guide to register alterations of normal morphophysiology during clinical, videoroscopy and morphological evaluations.

A diagram in which each anatomical region of the lip received a number was created in order to facilitate the registry of the lesion’s location (Fig. 1). This diagram attributed code numbers for each lip region, in order to register and map all clinical aspects identified by clinical and videoroscopy examination. The aspects of each region were documented and quantified. The Toluidine Blue Test (TBT) was performed as described by Epstein (2003) (7).

The biopsy site was determined after a thorough analysis of the data collected from all 14 areas of the lip as depicted in the diagram (Fig. 1). The site with the worst changes identified by physical examination, videoroscopy and the TBT was chosen for biopsy. In the case of any discrepancy among the three tests, the biopsy was performed at the site of injury with the greatest clinical severity. The histological sections were stained with both hematoxylin-eosin and periodic acid-Schiff stain, and the sections were analyzed under an optical microscope (Leica DM 2500, Leica Microsystems, Germany). The degree of epithelial dysplasia was determined based on the scale defined by the World Health Organization (8) and was categorized as mild, moderate or severe. Evaluation of the efficiency among the methods was performed using comparisons of proportions at a significance level of 5%. The SPSS 11.0 package was used for data analysis. Concordance was assessed by the McNemar test and X2. This study was submitted and approved by the Ethics Committee of our institution.

## Results

Of the 80 included patients, 45 (56.2%) were women and 68 (85%) were white. The age range of the patients was 10 to 86 years (MA= 55.1; MED= 56.0; SD=15.68). Biopsy was indicated and performed in a total of 52 patients. With regards to the location of AC, 70 patients (88.3%) presented with lesions exclusively on the lower lip. Twenty (25.0%) patients reported experiencing at least one symptom, whereas all patients (100%) presented with dryness of the lip, and 57 patients (71.2%) exhibited flaking ( Table 1).

**Table 1:**
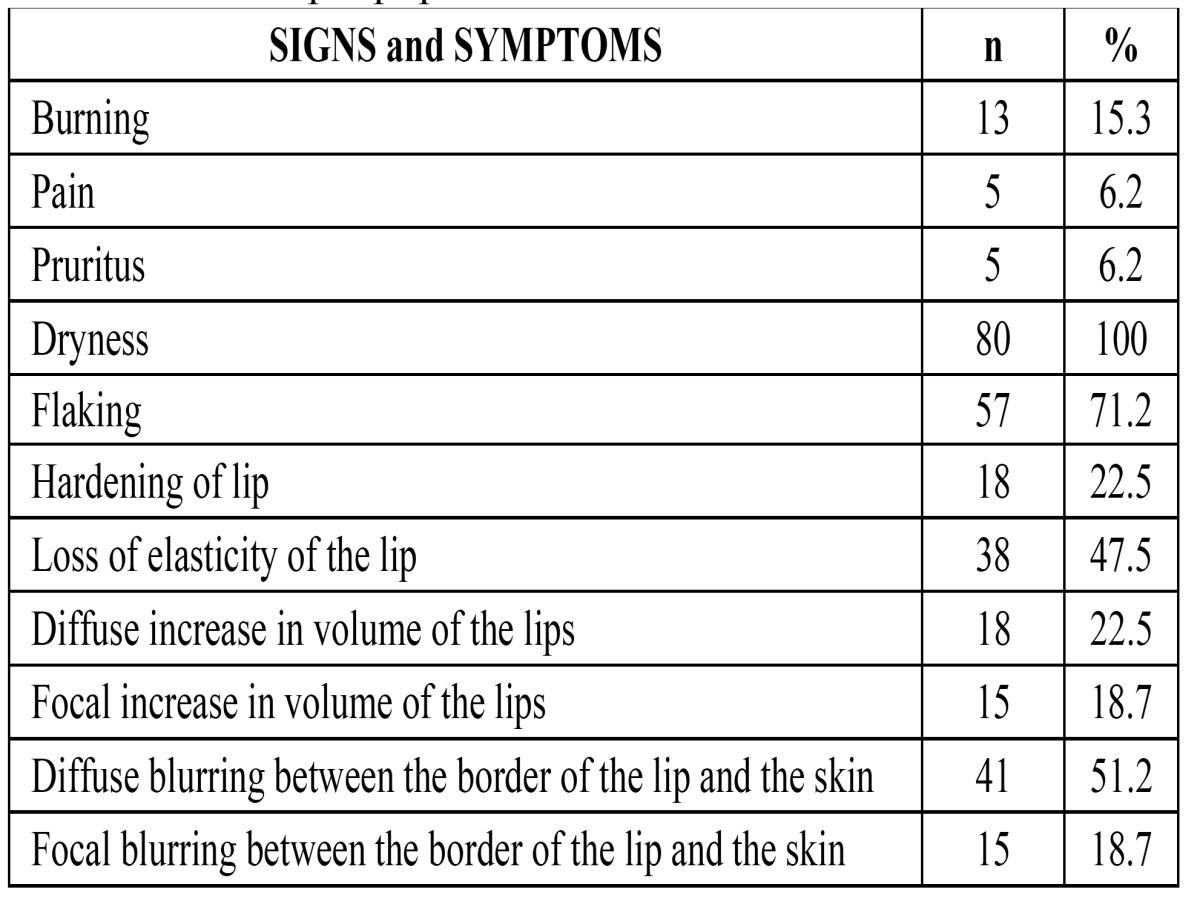
Signs and Symptoms identified and present in the patients of the sample population.

**Figure 2:**
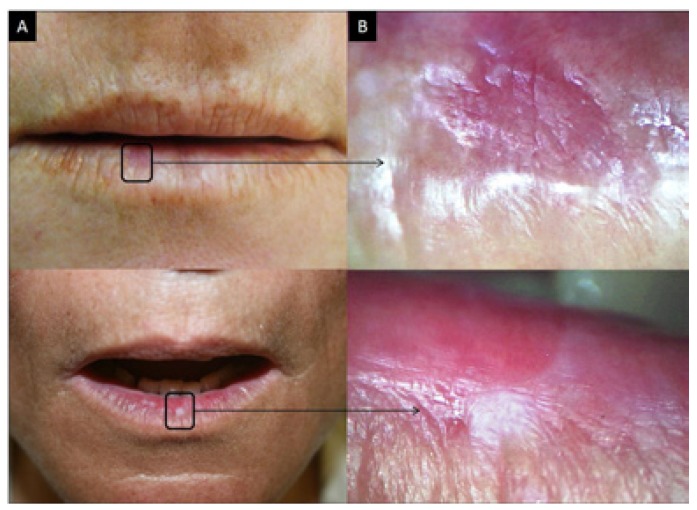
Actinic cheilitis. A. Photographic image; B. Videoroscopic image.

**Figure 3:**
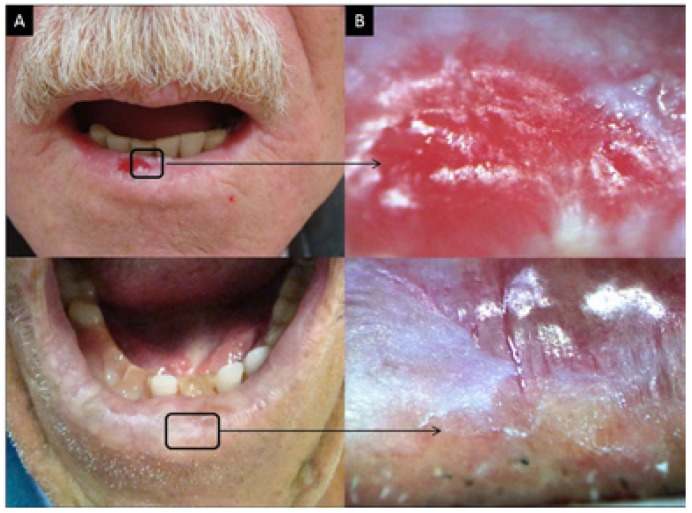
Actinic cheilitis. A. Photographic image; B. Videoroscopic image.

**Figure 4:**
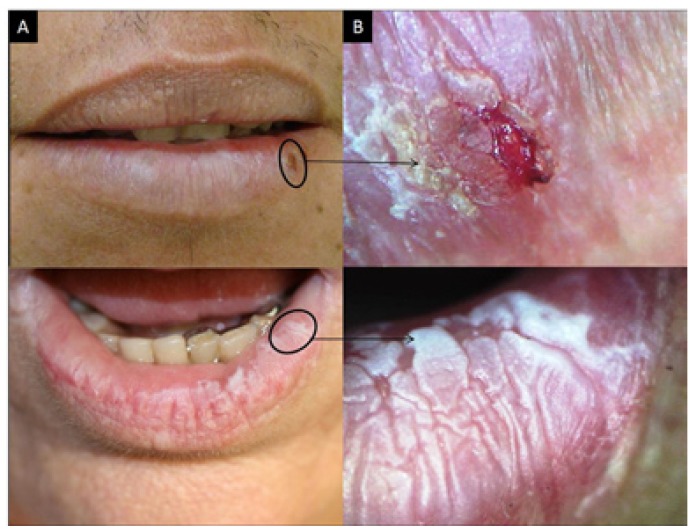
Actinic cheilitis. A. Photographic image; B. Videoroscopic image.

Although 1120 regions were examined using the diagram of the lip, only the results from the lower lip were considered when comparing the efficiency between oroscopy and videoroscopy methods because the lower lip was the most affected region. From the lower lip results, 720 regions were examined; a physical exam identified 278 lesions, and videoroscopy identified 557 lesions (Figs. 2A, 3A and 4A regarding oroscopy view, and Figs. 2B, 3B and 4B regarding videoroscopy view). Our primary findings obtained from the data collected by oroscopy paired with videoroscopy regarding the three most frequent lesions of the lower lip are as follows: 64/173 regions exhibited erythema, 92/214 regions contained white lesions, and 84/118 regions exhibited brown stains (focal or diffuse area with color alteration ranging from light to dark brown). These data exhibited a significant increase in the incidence of diagnosis (*p*<0.05) when videoroscopy was used (Table  Table 2). Although the absolute number of lesions encountered by videoroscopy was significantly higher than those encountered by oroscopy, the frequency of each type of lesion was not significantly different. Other lesions investigated accounted for less than 15% of the lesions found using either method. (Table  Table 2) The difference in the number of lesions found between the procedures was statistically significant for all lesions investigated except for ulcers and crust (Table  Table 3).

**Table 2:**
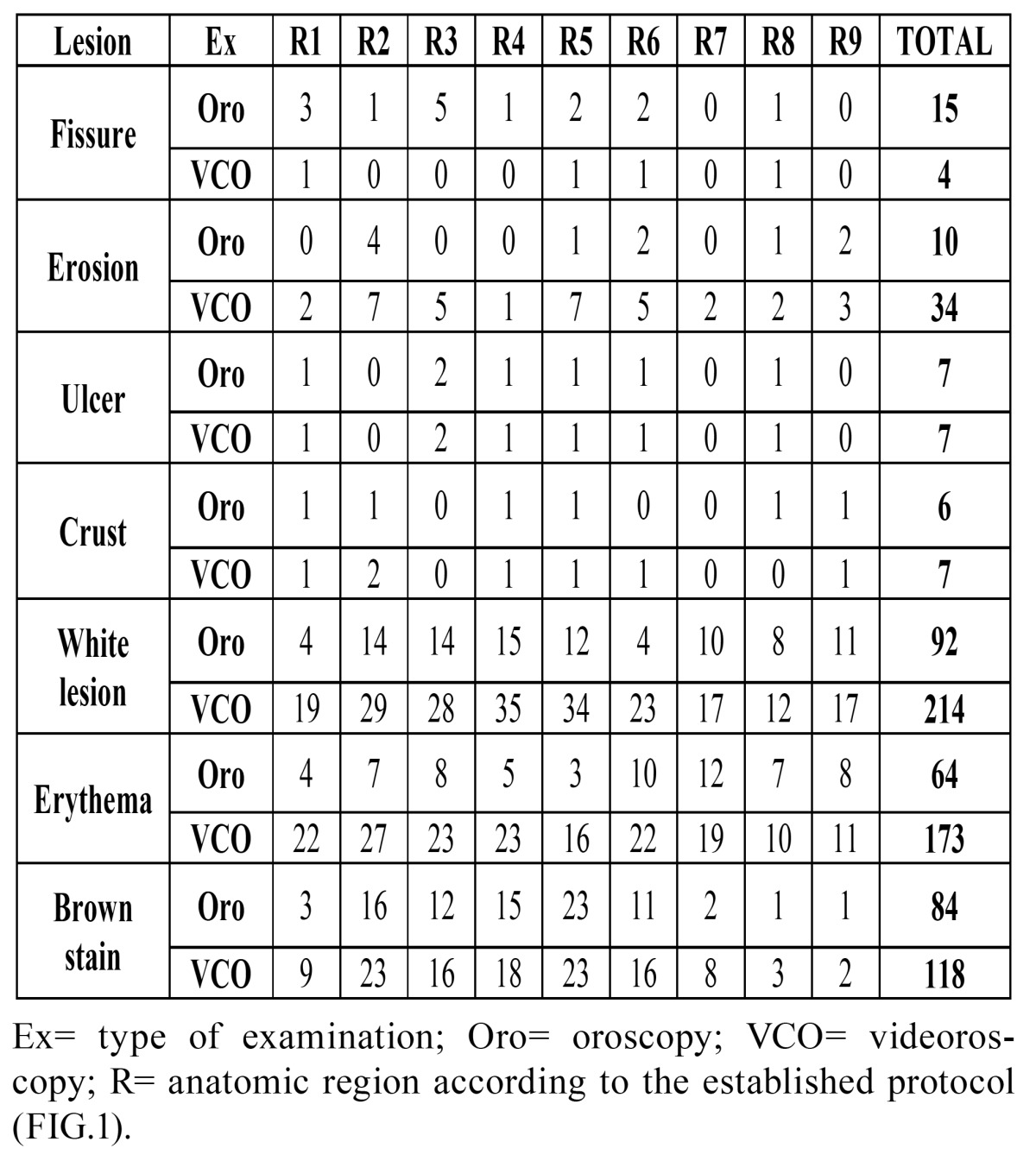
Distribution of the number and percentage of lesions identified in the lower lip by oroscopy (n = 278) and videoroscopy (n = 557) in 80 evaluated patients.

**Table 3:**
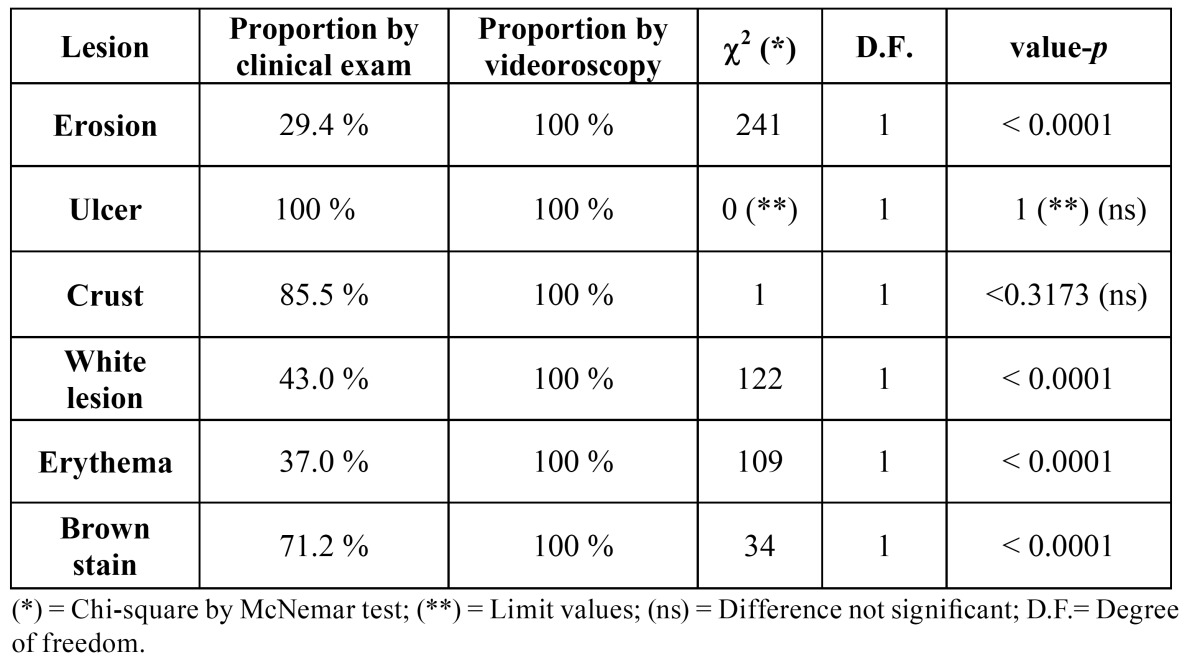
Comparison of the proportion of lesions identified by the videoroscopy method and the clinical exam.

The possible results for TBT test adopted in this study were positive, negative, and retention, and so we found 46 positive regions and 54 regions with retention ( Table 4). Among the 52 patients who underwent biopsy for initial diagnosis, six patients underwent more than one biopsy, and a total of 59 fragments were obtained and examined. From these 59 biopsy sites, 27 (45.7%) of the sites contained lesions that were identified by both oroscopy and videoroscopy; 12 (20.3%) of the lesion sites were identified by videoroscopy only, and videoroscopy was able to identify more lesions than oroscopy for 20 (33.9%) sites. From the 12 sites with lesions identified by videoroscopy only, the TBT was positive in 3 (25.0%) cases. New biopsies were indicated for 14 patients during follow up, and a total of 73 biopsies fragments were obtained. The main histopathological findings were as follows: 6 (8.2%) cases of AC were associated with carcinoma, and 65 (89.0%) cases of AC with epithelial dysplasia were identified ( Table 5). Among the 6 histopathologically identified carcinoma cases, TBT was positive in 4 (66.7%).

**Table 4:**
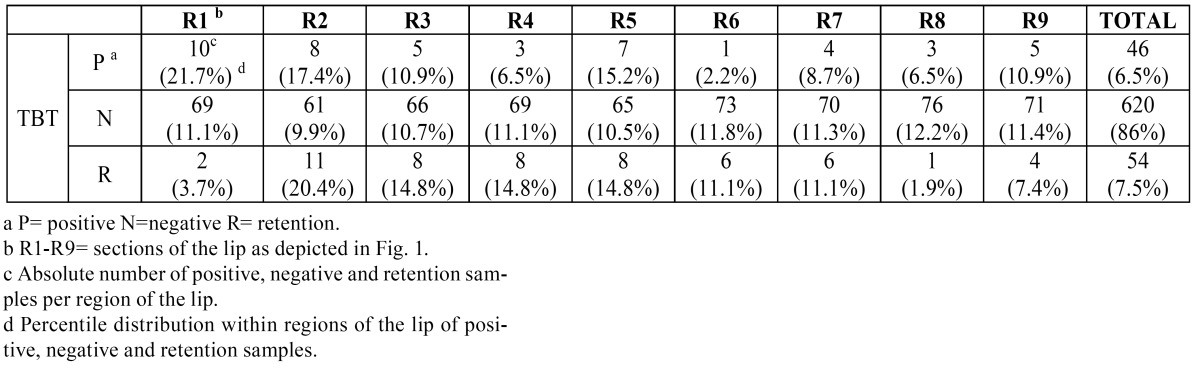
Distribution of positive, negative and retention samples identified in the lower lip by toluidine blue test in patient study samples.

**Table 5:**
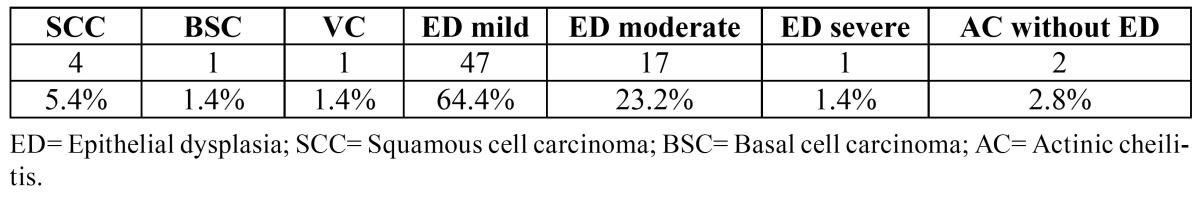
Distribution of histopathological results found in 73 biopsies.

## Discussion

Establishing the diagnosis of AC is important because the initial clinical manifestations are subtle. Furthermore, there is no correlation between clinical appearance and grade of histopathological damage, and there are no clear clinical aspects to distinguish AC from an early carcinoma. (5,6) The great difficulty in determining the most suitable site for biopsy is due to the diffuse aspect of the injury, which is often accompanied by subtle but important changes. (9) Most of the reports in the literature regarding the clinical changes in AC did not show quantitative and/or statistical analyses. In this study, the clinical features and the symptoms appeared at similar frequencies to those found in a previous study, which reported both quantitative and statistical analyses (3).

Currently, dermatoscopy or surface microscopy (as it was used in this study) is considered an important and relatively simple aid for daily clinical practice. (9-11) In this study, we performed a comparison of the results found by both physical examination and videoroscopy, and we observed that videoroscopy revealed a statistically higher frequency of incidence for most lesions. However, these results were not significantly different; thus, they are equally likely to be encountered by either method. Therefore, videoroscopy was able to correct discrepancies due to the limitations of oroscopy, such as in the case of fissures in which oroscopy identified more fissures than videoroscopy. For example, of the 15 fissures identified by oroscopy, 11 were only deeper grooves without the presence of erosion at the bottom of the groove, which is characteristic of fissures. In our experience, the use of videoroscopy permitted the detection of unobserved changes during physical examination that influenced the choice of the biopsy site and subsequently permitted the identification of a well-differentiated carcinoma before clinical evidence was present. In addition to its supremacy for lesion visualization and its contribution to determining the biopsy area, videoroscopy aided in these patients’ follow-up, and in some cases videoroscopy elucidated the reason behind positive toluidine blue testing in some regions where no changes were detected during the physical examination. The association of a positive TBT and a negative assessment via clinical examination could result in misinterpretation that this is a false positive test. Videoroscopy showed that all positive TBT patients presented with AC lesions and these were mainly associated with erosions in the cases in which no lesion was observed.

Generally, reports related to AC lesions exhibit a variety of degrees of dysplasia. In a previous study, 10.3% of the patients in the study group presented with mild dysplasia by histopathology, whereas 27.6% of the cases were of moderate dysplasia, and 62.1% of severe dysplasia. (3) In our casuistic, we demonstrated a greater number of histopathological findings with mild dysplasia. We believe that this observation was due to the early diagnosis achieved as a consequence of the use of the videoroscopy procedure.

Because 10 to 20% of AC cases progress to a well-established SCC, the appropriate monitoring of long-term changes found in the lip is very important. Nico *et al*. (2007) show a lack of correspondence between clinical and histopathological aspects, emphasizing the condition’s multi focal features (12). Because this type of lesion presents itself so variably, with no specific clinical features, and because for most people it seems harmless, the seriousness may be underestimated. The creation and use of the diagram of the lips proved to be a very useful tool and provided the possibility of recording each lesion with higher accuracy. The mapping also allowed for a more accurate record of the location of biopsies performed and follow-up of patients. Changes occur slowly, and if there is not an accurate record of the type of change and its exact location, the health professional may not realize that changes have occurred. We believe the use of the proposed diagram will increase the accuracy in diagnosis and thus avoid major mutilations. To our knowledge, this is the first time such tool has been reported.

## Conclusions

Because AC is a potentially malignant disorder, all possible auxiliary methods that contribute to an early diagnosis and efficient monitoring of the patient must be used. In our experience, the use of videoroscopy aided the detection of several lesions that had not been seen by the naked eye in patients with AC. The original diagram was useful to record the exact region where each lesion was found, thus facilitating patients’ follow-up.
